# Endothelial Neuropilin-1: a multifaced signal transducer with an emerging role in inflammation and atherosclerosis beyond angiogenesis

**DOI:** 10.1042/BST20230329

**Published:** 2024-02-07

**Authors:** Anissa Chikh, Claudio Raimondi

**Affiliations:** 1 Molecular and Clinical Sciences Research Institute, St. George's, University of London, London SW17 0RE, U.K.; 2 William Harvey Research Institute, Barts and The London School of Medicine and Dentistry, Centre of Cardiovascular Medicine and Devices, Queen Mary University of London, Charterhouse Square, London EC1M 6BQ, U.K.

**Keywords:** adherens junction, atherosclerosis, cadherins, endothelial cells, neuropilins, transforming growth factors

## Abstract

Neuropilin-1 (NRP1) is a transmembrane glycoprotein expressed by several cell types including, neurons, endothelial cells (ECs), smooth muscle cells, cardiomyocytes and immune cells comprising macrophages, dendritic cells and T cell subsets. Since NRP1 discovery in 1987 as an adhesion molecule in the frog nervous system, more than 2300 publications on PubMed investigated the function of NRP1 in physiological and pathological contexts. NRP1 has been characterised as a coreceptor for class 3 semaphorins and several members of the vascular endothelial growth factor (VEGF) family. Because the VEGF family is the main regulator of blood and lymphatic vessel growth in addition to promoting neurogenesis, neuronal patterning, neuroprotection and glial growth, the role of NRP1 in these biological processes has been extensively investigated. It is now established that NRP1 promotes the physiological growth of new vessels from pre-existing ones in the process of angiogenesis. Furthermore, several studies have shown that NRP1 mediates signalling pathways regulating pathological vascular growth in ocular neovascular diseases and tumour development. Less defined are the roles of NRP1 in maintaining the function of the quiescent established vasculature in an adult organism. This review will focus on the opposite roles of NRP1 in regulating transforming growth factor β signalling pathways in different cell types, and on the emerging role of endothelial NRP1 as an atheroprotective, anti-inflammatory factor involved in the response of ECs to shear stress.

## Introduction

Neuropilin-1 (NRP1) belongs to the Neuropilin family of receptors which includes NRP1 and NRP2. Both NRPs are transmembrane glycoproteins that share a 44% homology in their amino acid sequence and act as receptors for secreted peptides belonging to class 3 semaphorins and vascular endothelial growth factor (VEGF) families [1]. NRP1 is expressed by several cell types including endothelial cells (ECs) [2]. Originally identified as an adhesion molecule [3], NRP1 has been extensively studied as a VEGFA and semaphorin co-receptor in ECs and neurons [4] because of its key role in neurovascular development. Global and endothelium-specific NRP1 knockout mouse mutants die *in utero* with abnormal yolk sac and neuronal vascularisation [5,6]. Whereas the vascular defects of NRP1 knockouts were initially attributed to impaired VEGF signalling, several lines of evidence show that NRP1-dependent VEGFA signalling is not essential for vascular development [7,8]. It is now established that NRP1 regulates a range of signalling pathways in response to diverse ligands during angiogenesis. Similarly, in the quiescent vasculature, NRP1 interacts with several receptors and transmembrane proteins at the plasma membrane to regulate cellular responses [9]. For instance, recent evidence shows that in ECs NRP1 is part of mechanosensory complexes at the plasma membrane and that it plays a role in shaping the response to shear forces promoting atheroprotection [10]. In addition, we recently showed that a subcellular pool of NRP1 localises in the mitochondria and interacts with a mitochondrial iron transporter ABCB8 (ATP binding cassette subfamily B member 8) to regulate iron homeostasis and mitochondrial function in EC [11]. Thus, NRP1 has multifaceted roles in ECs signalling that profoundly affect EC and vascular functions.

## NRP1: domains and classical ligands

**Figure 1.**
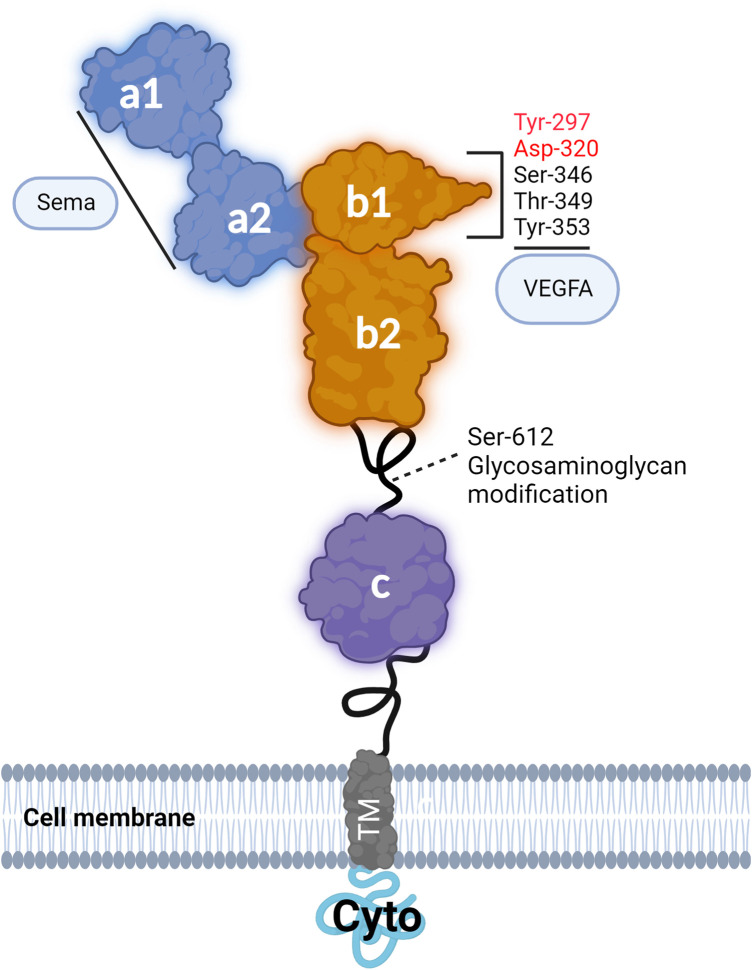
NRP1 structure and key amino acid residues of the extracellular domains mediating VEGF binding or undergoing post-translational modification. Schematic illustrating NRP1 structure and organisation in seven domains (a1, a2, b1, b2, c, TM, Cyto). The schematic shows the interactions of NRP1 with VEGFA and highlights the amino acid residues contributing to VEGFA binding or subjected to glycosaminoglycan modification. Highlighted in red are the amino acids in the b1 domain essential for VEGF binding.

**Figure 2.**
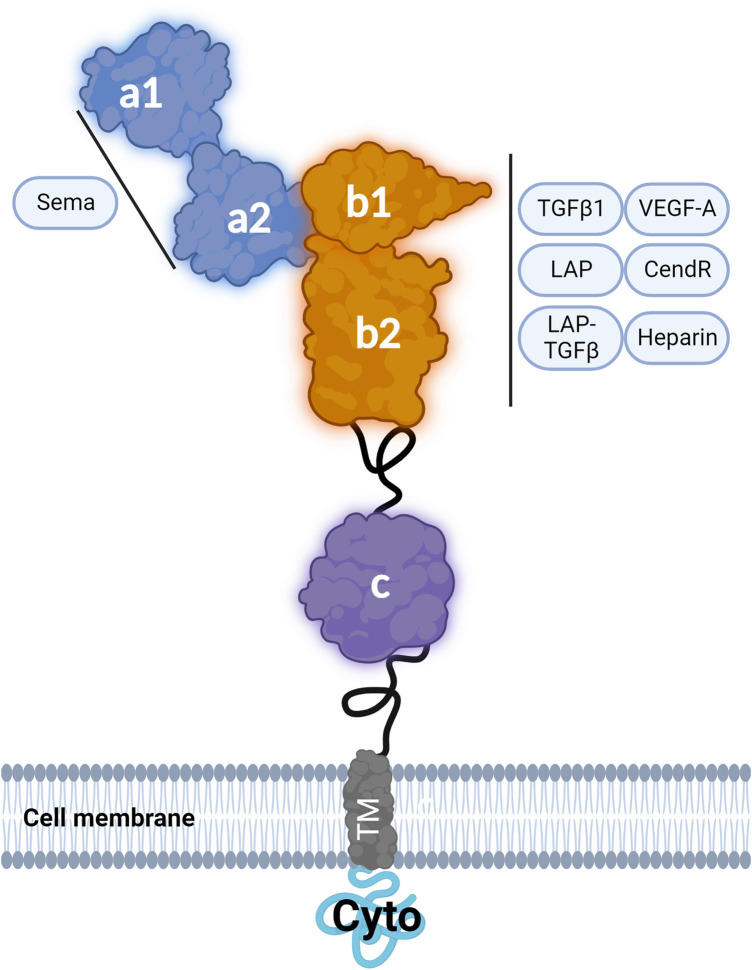
NRP1 structure and ligands. Schematic of NRP1 structure showing the interactions between the described NRP1 ligands and NRP1 protein domains.

NRP1 is a 130 kDa protein consisting of an extracellular moiety of ∼820 amino acids organised in five extracellular domains designated as a1, a2, b1, b2 and c. NRP1 has a transmembrane domain and a short intracellular domain (cyto) of ∼44 amino acids [9]. The intracellular cyto domain is catalytically inactive and has a SEA (Ser-Gln-Ala) motif which can interact and recruit intracellular proteins containing a PDZ (Postsynaptic density 95, Disk large, Zona occludens-1) domain such as the adaptor protein synectin (also known as GIPC1) [12]. The membrane-proximal extracellular c domain of NRP1 is known as the MAM domain due to the sequence similarities between the c domain and the analogous domains in meprin, A5, and mu-phosphatase (RPTPμ) [13]. Although no direct interaction between the MAM domain and other molecules has been demonstrated, this region may promote NRP1 dimerisation and multimerisation, which in turn modulates NRP1 complexes with other signalling receptors [9]. The a1 and a2 domains are members of the CUB domain structure family [13]. These domains are necessary for NRP1 binding to class 3 semaphorins (Sema3) [14]. The b1 and b2 domains, homologous to the coagulation factor (V/VIII) domain family, contain binding sites to growth factors including VEGFA [14,15] (Figures 1 and 2) and additionally contribute to the binding of a C-terminal arginine of class 3 semaphorins which have been cleaved by the proteolytic enzyme furin [16,17]. The b1 and b2 domains have been shown to interact also with VEGF homologues VEGFB, VEGFC and VEGFD, with heparin increasing the affinity of NRP1 to VEGFC [18–20] (Figure 1). Glycosaminoglycan modification occurs at residue Ser-612 of NRP1 with the attachment of heparan sulfate or chondroitin sulfate enhancing VEGF binding to NRP1 and VEGF signalling in ECs [21]. Whereas the b2 domain only partially contributes to VEGFA binding, the b1 domain is essential [14]. Structural biology studies have shown that the portion of VEGFA encoded by exon 7 of the *VEGFA* gene directly engages the L1 Loop of NRP1 b1 domain and that a cleft with a negative charge within NRP1 b1 domain interacts with the C terminal (Asp-Lys-Pro-Arg-Arg) region of VEGFA encoded by exon 8 of *VEGFA* gene [22,23]. The guanidinium moiety of Arg-164 of VEGFA C-terminus binds to NRP1 by forming a salt bridge with residue Asp-320 of NRP1 b1 domain while the carbonyl moiety forms hydrogen bonds with Ser-346, Thr-349 and Tyr-353 within the b1 domain [17,23] (Figure 1). In addition, the carbonyl backbone of the Lys residue in VEGFA C-terminus forms a hydrogen bond with Tyr-297 in NRP1 b1 domain [24] (Figure 1). Biochemical and functional studies have demonstrated that Asp-320 and Tyr-297 are critical for VEGFA binding to NRP1 with mutations of Asp-320 to Lys (D320K) or Tyr-297 to Ala (Y297A) abrogating VEGFA binding to NRP1 (Figure 1) without affecting NRP1 binding to class 3 semaphorins [7,8,25].

## NRP1 is a receptor for transforming growth factor β family members

In addition to VEGFs, NRP1 binds transforming growth factor β1 (TGF-β1), the amino-terminal domain of the TGF-β1 precursor peptide named latency associated peptide (LAP) and the latent complex formed by the binding of LAP with TGF-β1 [26] (Figure 2). In cell-free assays these ligands compete with VEGFA for NRP1 binding, suggesting that the interaction is mediated by the b1 and b2 domains. Competition experiments using a peptide containing the arginine-rich C-terminal motif of LAP showed that binding of this peptide to NRP1 blocked the binding of VEGFA and LAP-TGF-β1, suggesting the possibility that the C-terminal portion of LAP binds to the negatively charged cleft in the b1 domain as VEGFA [26]. Because active TGF-β1 lacks an arginine rich-C-terminal motif, it has been proposed that a basic motif encompassing Arg-94 of TGF-β1 (Arg-Lys-Pro-Lys), shown to mediate the interaction with TGF-β receptor 2 [27,28], could bind to NRP1 electronegative pocket of the b1 domain. NRP1 has been shown to activate TGF-β1 from its LAP-bound form through an uncharacterised mechanism [26]. It has been anticipated that a basic sequence (Arg-Lys-Phe-Lys) in NRP1 b2 domain binds to the N-terminal portion of LAP [26], activating latent TGF-β1 through a mechanism similar to that by which an Arg-Phe-Lys sequence of thrombospondin-1 activates TGF-β [29]. Experimental work is required to verify the proposed mechanism but together this evidence strongly indicates that NRP1 b domains mediate TGF-β binding.

## NRP1 interacts with TGF-β receptors

TGF-β isoforms (TGF-β1, TGF-β2, TGF-β3) signal by inducing oligomerisation of two type I TGF-β receptors (ALK5 or ALK1) with two molecules of type II TGF-β receptor (TGFBR2), forming a heterotetrameric complex [30–32]. Within this complex, TGFBR2 phosphorylates the type I receptors in the (Thr-Thr-Ser-Gly-Ser-Gly-Ser-Gly) GS domain of the juxtamembrane domain inducing activation of the type I receptor kinase activity [33]. The activated type I receptor induces a downstream ‘canonical’ pathway involving the phosphorylation of the cytoplasmic proteins SMAD2 and SMAD3 [34]. Phosphorylated SMAD2 and SMAD3 bind SMAD4 and translocate into the nucleus to mediate gene transcription [34]. TGF-β receptors can also initiate a ‘non-canonical’ SMAD-independent signalling pathway that results in the activation of Rho-GTPases, PI3K/AKT, RAS-dependent ERK1/2 phosphorylation, TAK1-dependent NFkB, p38 and JNK pathways [35]. In cell-free assays, NRP1 has been shown to interact with high affinity with TGF-β type I and II receptors, with a higher relative affinity for ALK5 than for TGFBR2 [36]. Under these experimental conditions, the binding of NRP1 to type I TGF-β receptor is not altered by the presence of TGF-β1, whereas TGF-β1 increases NRP1 binding to TGFBR2 [36]. In addition, NRP1 binds and bridges type I and II receptors independently of TGF-β1 and facilitates binding of TGF-β to the receptor complex [36]. Together this evidence indicates that NRP1 could modulate TGF-β signalling at different levels: (1) by binding TGF-β, LAP-TGF-β1 and activating TGF-β, NRP1 could enhance TGF-β availability potentially improving the presentation of TGFβ ligands to TGF-β receptors; (2) by binding to the complexes of TGF-β receptors, NRP1 could increase the affinity of the receptors towards TGF-β and promote TGF-β receptors activation and downstream signalling.

## NRP1 acts as a positive or negative regulator of TGF-β in a cell-specific manner

**Figure 3.**
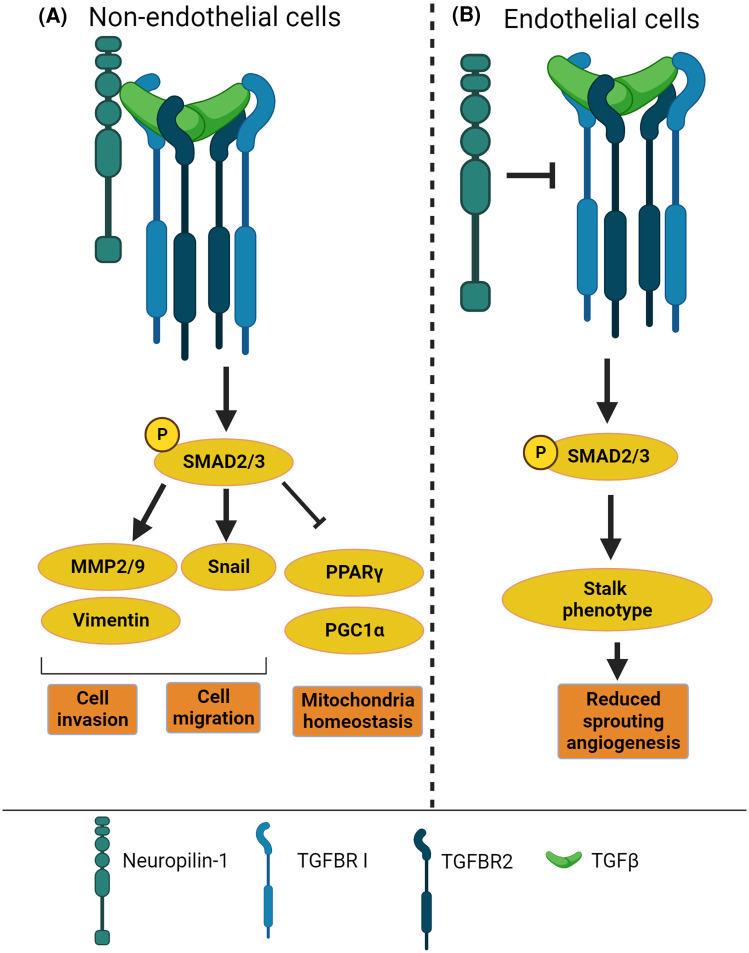
NRP1 regulation of TGF-β signalling is cell-specific. (**A**) Schematic diagram showing the role of NRP1 in activating TGF-β signalling and downstream effectors in non-ECs to modulate cell invasion, cell migration and mitochondria homeostasis. (**B**) Diagram describing that NRP1 promotes sprouting angiogenesis by acting as an inhibitor of TGF-β signalling, which induces a stalk phenotype [39] characterised by decreased cell migration, therefore reducing sprouting angiogenesis.

In breast cancer cell lines MDA-MB-231, MCF7 and MDA-MB-435, NRP1 promotes TGF-β canonical signalling modulating SMAD-dependent transcriptional activity and cell proliferation [36] (Figure 3A). In non-small cell lung cancer cell lines, NRP1 promotes canonical SMAD-dependent signalling inducing cell migration, matrigel invasion and epithelial-mesenchymal transition [37]. Also, in T cells, NRP1 mediates TGF-β1-dependent conversion of T effector cells into T regulatory cells [26,37] although it has not been determined whether by contributing to TGF-β canonical or non-canonical pathways. In smooth muscle cells and cardiomyocytes, NRP1 down-regulation reduces TGF-β canonical pathway, with loss of NRP1 expression in these cell types decreasing mouse survival and correlating with cardiomyopathy [38]. Whereas this evidence supports a widespread role of NRP1 as a positive regulator of TGF-β in mesenchymal, immune and cancer cells (Figure 3A), in ECs NRP1 acts as a negative regulator of TGF-β signalling (Figure 3B). Silencing *in vitro* NRP1 gene expression in ECs enhances baseline and TGF-β-induced phosphorylation of SMAD3 [39]. Accordingly, the developing brain of endothelial-specific NRP1 knockout shows higher levels of phosphorylated SMAD3 in ECs compared with littermate controls [40]. Mechanistically, in the neuro-vasculature, NRP1 forms intercellular protein complexes in trans with Integrin β8 to promote vascular/neuroepithelial cell adhesion in the developing brain [40]. Similarly, postnatal analysis of the vasculature in the mouse retina, which represents an excellent model to visualise and quantify vascular growth during postnatal angiogenesis, shows that NRP1 in ECs promotes angiogenesis by inhibiting canonical TGF-β signalling through a mechanism that requires NRP1 extracellular and transmembrane domains but not the cytoplasmic domain [39]. The surprising opposing roles of NRP1 in the regulation of TGF-β signalling in ECs versus smooth muscle, cardiomyocytes, immune and cancer cells remain to be fully elucidated. However, it could be speculated that the different repertoire of NRP1 binding partners expressed by ECs in the plasma membrane compartment determines whether NRP1 activates or inhibits TGF-β.

## Vascular endothelial-cadherin regulates TGF-β signalling

ECs express vascular endothelial cadherin (VE-cadherin), a classical cadherin [41] encoded by the *CDH5* gene located in chromosome 16. VE-cadherin is EC restricted [41] and presents five extracellular cadherin domains that mediate cell-cell adhesion via calcium-dependent homophilic interactions forming a type of cell-cell junction named adherens junction (AJ) [42]. VE-cadherin has a cytoplasmic domain which interacts with adaptor molecules belonging to the catenin family (α catenin, β catenin, p120 catenin), which stabilise AJs by coupling VE-cadherin to the actin cytoskeleton and by inhibiting VE-cadherin internalisation [43–45]. AJs mediate intercellular adhesion between ECs and regulate key aspects of vascular function in the quiescent vasculature such as vascular permeability [46] and transmigration of circulating leukocytes across the endothelium [47,48]. In addition, VE-cadherin regulates transduction of several signalling pathways. For instance, VE-cadherin mediates contact inhibition signals by interacting with VEGFR2 and limiting VEGFR2 endocytosis and downstream signalling via MAPK [49]. VE-cadherin promotes actin cytoskeleton contractility via Rho-kinase-dependent myosin light-chain 2 phosphorylation and cytoskeleton dynamics by promoting Rac1 activation and phosphorylation of p21-activated-kinase [50]. Furthermore, by binding p120 catenin, VE-cadherin localises at cell-cell junctions the activation of Rho GTPases including Cdc42 and Rac1 mediated by p120 catenin [51,52]. Relevant to NRP1 signalling, VE-cadherin interacts with type I TGF-β receptors ALK1, ALK5, type II TGF-β receptor TGFBR2 and Endoglin [53]. Endoglin is a 90 kDa transmembrane glycoprotein expressed by ECs that acts as an auxiliary co-receptor for TGF-β family ligands [54]. Following TGF-β stimulation of ECs, a subset of activated TGF-β receptors co-localises with VE-cadherin at AJs promoting TGF-β signalling [53]. Accordingly, in ECs, VE-cadherin is required for effective TGF-β-induced phosphorylation of SMAD2 downstream of ALK1 and ALK5 [53]. Thus, VE-cadherin is a positive regulator of TGF-β, unique to ECs, that functionally connects AJs to TGF-β signalling.

## Signalling cross-talk mediated by NRP1

**Figure 4.**
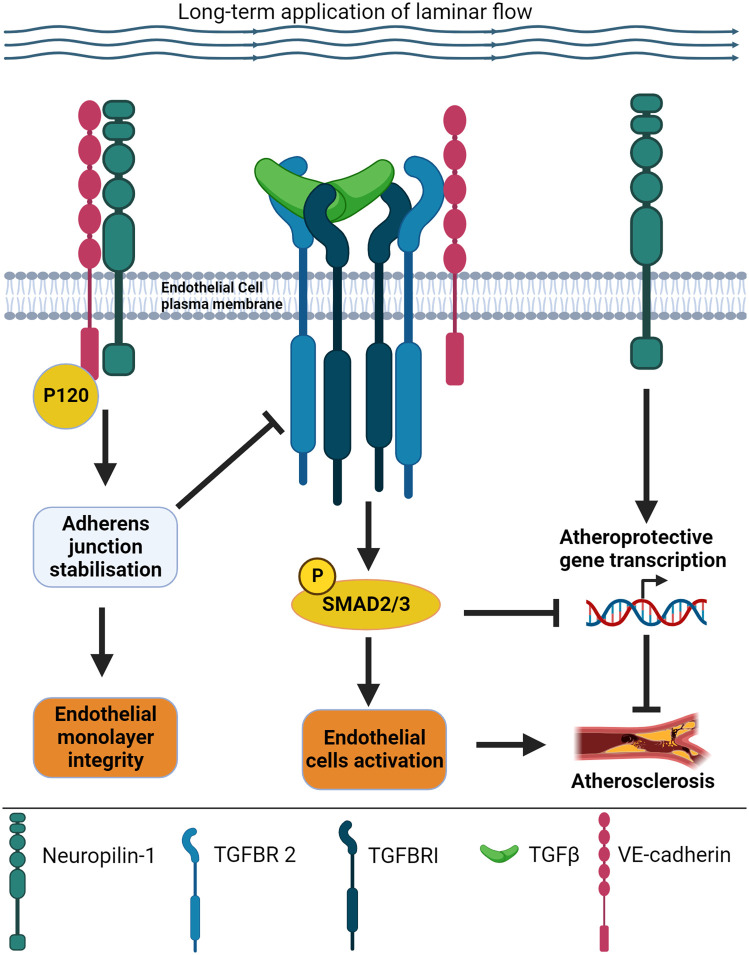
Schematic illustration of the working model intertwining NRP1, VE-cadherin and TGFBR2 to regulate TGF-β signalling. NRP1 interacts with VE-cadherin and p120 catenin (P120) leading to adherens junction stability and endothelial monolayer integrity in cells exposed to laminar flow shear stress for 24 h. By promoting adherens junction stability, NRP1 suppresses phosphorylation of SMAD2/3 downstream of TGF-β receptors, preventing endothelial activation. Under laminar flow, NRP1 prevents the transcription of pro-inflammatory genes, reducing the interaction between ECs and inflammatory cells and preventing atherosclerosis.

In physiological conditions, ECs are in direct contact with the circulating blood and exposed to linear flow along straight vessels, or turbulent flow at vessel branches and bends [55]. The flowing blood induces the mechanical force shear stress, which is detected by protein sensors located on the EC cell surface. These sensors then translate the force into intracellular biochemical signals which drive changes in cell function by affecting morphology, gene expression, and metabolism [55]. In experimental models, ECs exposed to laminar ‘atheroprotective’ flow elongate and align with the direction of flow, resembling the alignment observed in straight blood vessels in the body [56]. Alignment is accompanied by the expression of anti-inflammatory genes and ‘healthy’ EC function [56]. In contrast, ECs exposed to turbulent ‘atherogenic’ flow do not align, show pro-inflammatory characteristics and ‘unhealthy’ function [56,57]. Regions of turbulent flow in vessels are the major sites of atherosclerosis, the pathological process that narrows blood vessels, reducing oxygen and nutrient delivery to tissues. Recent evidence shows that the plasma membrane pool of NRP1 colocalises with VE-cadherin at cell-cell adhesion sites where it interacts with VE-cadherin [58,59]. NRP1 modulates AJs dynamics in ECs cultured in static conditions [58] and AJs stability in ECs exposed to laminar flow [59]. Accordingly, in static conditions, ECs knockdown for NRP1 display larger VE-cadherin-containing AJs and reduced VE-cadherin turnover at AJs [58] whereas down-regulation of NRP1 in human umbilical vein ECs (HUVECs) exposed to laminar flow causes the weakening of cell-cell contacts and the appearance of gaps between adjacent ECs [59]. Supporting a role of NRP1 in stabilising AJs in ECs exposed to laminar flow, loss of NRP1 in the descending aortic endothelium induces a discontinuous VE-cadherin distribution and the appearance of finger-like protrusions between adjacent ECs, consistent with weaker AJs [59]. Mechanistically, NRP1 forms a complex with VE-cadherin and p120 catenin with NRP1 binding to VE-cadherin promoting the interaction of VE-cadherin with p120 [59] (Figure 4). The destabilisation of AJs by down-regulating NRP1 or p120 catenin increases the plasma membrane localisation of TGFBR2, SMAD2 phosphorylation and the activation of the TGF-β canonical pathway [59] (Figure 4). This evidence indicates that by binding VE-cadherin and stabilising the VE-cadherin adhesion complex, NRP1 restrains TGF-β signalling via VE-cadherin (Figure 4). Furthermore, these data indicate that the endothelial-specific expression of VE-cadherin could explain the opposing roles of NRP1 towards TGF-β signalling in ECs versus cells devoid of VE-cadherin, with VE-cadherin adding a further layer of regulation to TGF-β signalling specific to ECs. In support of this idea, the destabilisation of AJs by down-regulating p120, which results in increased VE-cadherin endocytosis and turnover [43–45], diminishes the interaction of NRP1 with TGFBR2 resulting in increased TGFBR2 membrane localisation and SMAD2 phosphorylation [59]. To shed further light on the molecular mechanism intertwining NRP1, TGFBR2 and VE-cadherin, it remains to determine whether NRP1, VE-cadherin and TGFBR2 form a trimeric complex and whether the formation of this complex modifies NRP1-dependent TGF-β functional outputs. Several, not mutually exclusive, scenarios could be hypothesised: (1) In ECs, the function of NRP1 in inhibiting TGF-β signalling downstream of VE-cadherin by stabilising AJs is more prominent compared with the autonomous function of NRP1 to act as a TGF-β coreceptor and activator of TGF-β signalling, resulting in a net inhibition of TGF-β signalling in the endothelium; (2) VE-cadherin interaction with NRP1 could prevent the binding of TGF-β ligands and TGF-β receptors to NRP1 or induce conformational changes decreasing the affinity of NRP1 for TGF-β ligands and TGF-β receptors, thus reducing the overall capacity of NRP1 *per se* to promote TGF-β signalling; and (3) NRP1 could affect VE-cadherin-dependent TGF-β signalling by modulating the trafficking of the VE-cadherin/TGFBR2 signalling complex. Accordingly, in ECs, under static conditions, NRP1 down-regulation reduces the fluorescence recovery after photobleaching of fluorescent exogenous VE-cadherin at the plasma membrane [58], indicating that NRP1 regulates VE-cadherin turnover (and potentially turnover of protein complexes formed by VE-cadherin), either by promoting recovery to the plasma membrane from the endocytic pathways or by reducing VE-cadherin endocytosis. This would be consistent with the established role of NRP1 in promoting VEGFR2 signalling by mediating VEGFR2 trafficking [60,61]. Accordingly, transcriptional changes affecting endosome trafficking pathways were observed in NRP1 knockdown ECs [59].

## NRP1 involved in inflammation and atherosclerosis

Stimulation of ECs with TGF-β induces expression of pro-inflammatory genes. Intradermal injection of TNFα into the ear of endothelial-specific mice expressing ALK5 or TGFBR2 conditional alleles shows reduced expression of the pro-inflammatory adhesion molecules ICAM-1 and VCAM-1 and leukocyte infiltration compared with controls, indicating that TGF-β drives endothelial inflammation and activation [62]. Accordingly, endothelial deletion of ALK5 or TGFBR2 in the ApoE^−/−^ atherosclerosis mouse model reduces atherosclerosis plaque burden, plaque collagen content, fibronectin deposition and VCAM-1 expression [62]. Inflammatory stimuli such as TNFα down-regulate NRP1 gene and protein expression [63], indicating that the inflammatory response in ECs down-regulates NRP1 which in turn leads to increased TGF-β signalling [59]. In agreement, recent gene transcription profiling shows increased expression of several inflammatory genes in ECs knockdown for NRP1, including genes encoding TNF superfamily members, C-reactive protein and pro-inflammatory cytokines belonging to the interleukin family (e.g. IL6, IL11 and IL12) and TGF-β family members (e.g. TGF-β1, TGF-β2 and TGF-β3) [59] (Figure 4). These data indicate that in addition to suppressing TGF-β signalling, NRP1 also suppresses the expression of TGF-β isoforms. Thus, in ECs, NRP1 regulation of TGF-β is twofold: (1) NRP1 suppresses TGF-β signalling by stabilising VE-cadherin at cell-cell contacts [59]; and (2) NRP1 inhibits the expression of TGF-β ligands, thus controlling an autocrine TGF-β pathway that drives an inflammatory response [59] in the endothelium. Functionally, NRP1 down-regulation in the endothelium increases endothelial activation and inflammation, up-regulating the expression of inflammatory adhesion proteins such as VCAM-1 and inducing leukocyte-endothelial interaction [59] (Figure 4). This correlates with increased atherosclerotic plaque in an ApoE atherosclerosis mouse mutant lacking NRP1 expression in the endothelium, indicating a key anti-inflammatory and atheroprotective role of endothelial NRP1 [59].

## NRP1 influences EC response to shear stress

**Figure 5.**
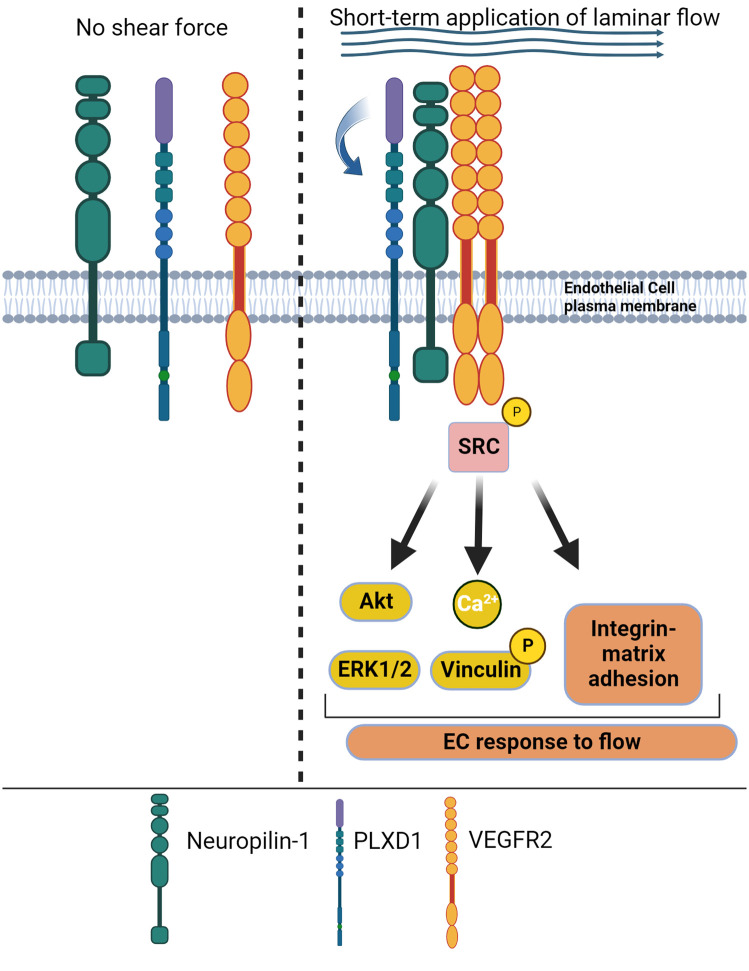
NRP1 forms a mechanosensory complex with PLXD1 and VEGFR2. Short-term application of laminar flow shear stress (2–5 min) induces the assembly of a mechanosensory complex formed by NRP1, PLXD1 and VEGFR2. NRP1 is essential to promote the formation of the complex and PLXD1 acts as the mechanosensor, with flow shear stress inducing flexion and conformational changes of PLXD1 ectodomain (curved arrow). Activation of the mechanosensory complex induces phosphorylation of VEGFR2 and downstream signalling, promoting EC response to flow.

ECs are equipped with mechanosensory complexes on the plasma membrane at the cell-cell junction interface formed by VEGFR2, VE-cadherin and the adhesion protein PECAM-1 that transduces shear stress [64]. Furthermore, transmembrane receptors of the integrin family transmit mechanical stresses from the extracellular matrix to the cytoskeleton across the plasma membrane, mediating downstream signal transduction [65]. Recent work has shown that in ECs, NRP1 forms a multimeric mechanosensing complex with the semaphorin receptor PlexinD1 (PLXND1) and VEGFR2 [10] (Figure 5). The PLXND1/VEGFR2/NRP1 complex is required for PLXND1-dependent response to laminar flow shear stress, independently of VE-cadherin and PECAM1, and it is sufficient to confer mechanosensitivity [10]. Thus, these findings suggest that the PLXD1/NRP1/VEGFR2 complex operates upstream of the junctional complex in response to shear stress. The PLXD1/NRP1/VEGFR2 complex transduces shear stress signalling in response to short-term exposure to laminar flow shear stress (2 min) inducing downstream phosphorylation of VEGFR2 and association of VEGFR2 with Src kinases in a mechanism requiring the presence of NRP1 and the flexion of the PLXND1 ectodomain [10] (Figure 5). A more recent study investigated the effect of long-term exposure to laminar flow shear stress (24 h) on NRP1 expression and the role of NRP1 in modulating transcriptional response to shear stress [59]. This work showed that long-term exposure to shear stress *in vitro* increases NRP1 expression in HUVECs and that NRP1 promotes shear stress-dependent alignment to the direction of flow in ECs exposed to atheroprotective laminar flow [59]. Down-regulation of NRP1 had no effect on the induction of established shear stress-induced genes such as Kruppel-like factor 2 (KLF2) and KLF4 [66,67], indicating that NRP1 is dispensable to the mechanosensor-dependent transcriptional response induced by long-term exposure to flow. However, comparison of transcriptional profiles of HUVECs expressing or lacking NRP1 and exposed to atheroprotective, atherogenic flow patterns or cultured in the absence of flow, showed that NRP1 down-regulation modulates flow-dependent transcriptional changes involved in cell adhesion, proliferation, oxidative stress, and inflammation independently of typical flow-induced genes such as KLF2 and KLF4 [59]. The new evidence indicates a bi-directional functional relationship between NRP1 and shear stress, with exposure to shear stress increasing NRP1 expression to promote the atheroprotective, anti-inflammatory effect of atheroprotective laminar flow. Together, these new lines of evidence highlight NRP1 as a key player in promoting EC and vascular homeostasis.

## Challenges of targeting specific NRP1 functions

In ECs, NRP1 modulates several signalling pathways downstream of several growth factors, cell-cell and cell-matrix contacts (Figures 2–5). Because originally NRP1 has been established as a key regulator in EC of VEGFA signalling, many approaches have been used to inhibit NRP1 binding to VEGFA as a potential therapeutic target to prevent angiogenesis in cancer and neovascular eye diseases. Small peptides sharing the Arg/Lys/X-X/Arg/Lys motif with the C-terminal domain of VEGFA isoform VEGFA-165 (named C-end-Rule or CendR) have been shown to compete with VEGFA for NRP1 binding and to inhibit NRP1-dependent VEGF signalling [68] (Figure 2). These peptides include heptapeptide A7R (ATWLPPR) [69,70], the immunostimulatory peptide Tuftsin [71] and peptides containing the RGD integrin recognition motif [72]. The small molecule EG00229 was designed to interact with the VEGFA binding pocket of NRP1, based on a bicyclic peptide corresponding to the last 28 C-terminal amino acids of VEGFA-165 [73]. EG00229 has anti-tumoral activity in different tumours, inhibiting NRP1-dependent endothelial and tumour cell migration downstream of VEGFA [73,74]. Furthermore, EG00229 synergises with the anti-cancer kinase inhibitor Lenvatinib in cholangiocarcinoma cells [75] and increases the cytotoxic effect of 5-fluorouracil and paclitaxel in lung cancer cells [73]. Because of its promising anti-cancer properties, EG00229 served as a lead compound to develop new NRP1 inhibitors such as EG01377 [76]. In addition to small molecule inhibitors, monoclonal antibodies have been generated against the b1 domain to block VEGFA binding to the b1. Genentech developed an anti-NRP1 monoclonal antibody [77] generated with an immunogenic sequence encompassing b1 and b2 domains that inhibits VEGF signalling with additive efficacy when combined with the anti-VEGFA humanised antibody Bevacizumab. The antibody failed in Phase I clinical trial because of severe proteinuria in over 50% of subjects [78]. More recently, an open-label, multicentre, Phase I clinical trial tested the safety of CEND-1, a novel cyclic peptide targeting αV integrins and NRP1 in patients with metastatic pancreatic ductal adenocarcinoma in combination with nab-paclitaxel and gemcitabine [79]. The study showed that CEND-1 in combination with nab-paclitaxel and gemcitabine has an acceptable safety profile, with adverse events generally consistent with those seen with nab-paclitaxel and gemcitabine [79]. The knowledge acquired in the last fifteen years of NRP1 research showed that in addition to VEGFA other ligands bind to the b domains with some ligands sharing binding sites for the b1 domain (e.g. VEGFA and TGF-β1) and that NRP1 interacts with multiple transmembrane receptors via its extracellular domains. Thus, targeting NRP1 binding pocket in the b1 domain could result in the modulation of multiple NRP1 functions beyond the intended targeted function. For instance, the binding of an arginine-rich C-terminal peptide (Gln-Ser-Ser-Arg-His-Arg-Arg) to the b1 domain competes with VEGFA, TGF-β1 and LAP binding [26]. Thus, particular attention should be given to test the effect of blocking peptides, small molecules and antibodies targeting NRP1 on all the different NRP1-dependent pathways since NRP1 exerts different independent functions, with sometimes surprising opposite roles depending on the cell type. The unintentional targeting of multiple NRP1-dependent pathways could explain the toxicity and side effects observed during the clinical trial with the Genentech anti-NRP1 monoclonal antibody, since the indiscriminate targeting of NRP1 functions could compromise homeostatic pathways in tissues and in the vascular system. For instance, binding of peptides, small molecules and antibodies could interfere with the atheroprotective function of NRP1 by interfering with its role in stabilising AJ stability and regulating downstream signalling. Accordingly, upon binding of CendR tetramers or following treatment with a blocking antibody directed against VEGFA–NRP1 binding, NRP1 undergoes redistribution at cell-cell contacts followed by internalisation [80]. This event results in vascular leakage through a mechanism involving the NRP1 cyto domain but independently of VEGFR2 activation [80]. In addition, recent evidence shows that NRP1 at the plasma membrane regulates mechanosensing and AJ signalling, suppressing signals involved in inflammation and atherosclerosis (Figures 4 and 5). Consequently, the overall effect of targeting NRP1 on the atheroprotective and anti-inflammatory function of NRP1 should be considered and investigated as the unintended inhibition of these functions would be detrimental for tissue and vascular homeostasis. Furthermore, structural and functional studies aimed at identifying strategies to target the binding of specific ligands and the interaction with receptors and adhesion molecules will be instrumental in empowering the design of strategies to specifically block or stimulate single NRP1 functions in physiopathological contexts.

## Perspectives

- Research in the last 10 years has shown that NRP1 has more functions in addition to the ‘canonical’ roles as a semaphorin and VEGF receptor.
- The recent discoveries of the role of NRP1 in promoting AJs stability, atheroprotection and in suppressing TGF-β signalling and inflammation in ECs (Figures 3 and 4), together with its active role in mechanotransduction (Figure 5) and in modulating EC response to shear stress, highlight NRP1 as an important factor to maintain endothelial and vascular homeostasis.
- The functional overlapping of NRP1 protein domains in the binding of several NRP1 ligands (Figure 2) and in promoting the interactions with ubiquitous or cell-type-specific cellular binding partners (i.e. VE-cadherin; Figure 4), poses a challenge in devising strategies to inhibit specific functions of NRP1 without interfering with other NRP1-dependent pathways.

## Abbreviations

AJ: adherens junction
EC: endothelial cell
HUVEC: human umbilical vein EC
KLF2: Kruppel-like factor 2
KLF4: Kruppel-like factor 4
LAP: latency associated peptide
NRP1: Neuropilin-1
NRP2: Neuropilin-2
PLXND1: PlexinD1
TGF-β1: transforming growth factor β1
VE-cadherin: vascular endothelial cadherin
VEGF: vascular endothelial growth factor
